# Metastatic Melanoma to the Urinary Bladder of Ocular Origin Accompanied with Primary Cutaneous Melanoma: Diagnostic Challenge—A Report of a Case

**DOI:** 10.1155/2017/4818537

**Published:** 2017-09-11

**Authors:** Constantine Theocharides, Kyriakos Chatzopoulos, Dimitrios Papanikolaou, Vasileios Siokas, Ioannis Amplianitis, Athanasios Papanikolaou

**Affiliations:** ^1^Department of Pathology, “Gennimatas” General Hospital, Thessaloniki, Greece; ^2^Department of Urology, Papageorgiou General Hospital, Thessaloniki, Greece; ^3^Department of Pathology, “Hippokration” General Hospital, Thessaloniki, Greece

## Abstract

Metastases of melanoma to the urinary bladder are infrequent. Even rarer are metastases to the urinary bladder from uveal melanoma, with only 3 cases published in the literature so far. Herein we present a case of a 77-year-old male patient who presented with metastatic melanoma to the urinary bladder. The patient's history included the diagnoses of uveal melanoma treated with radiation 25 years ago, as well as that of cutaneous melanoma diagnosed 7 years ago. The molecular study of the urinary bladder tumor specimen identified mutation of the GNAQ gene, which has been suggested to be an early molecular event in the pathogenetic course of over 80% of uveal melanomas. Therefore, the diagnosis of uveal melanoma metastatic to the urinary bladder was made.

## 1. Introduction

The metastatic tumors to urinary bladder are extremely rare [1–5]. Given the infrequency of the metastatic tumors to the urinary bladder and due to the fact that malignant melanomas consist of a subgroup of the specified tumors, metastatic melanoma to urinary bladder is designated an even more rare histopathological entity. The fact that primary malignant melanoma of urinary bladder is also a very rare lesion [6–8] must also be taken into consideration in the differential diagnosis of a melanocytic lesion of the urinary bladder [9–11].

Metastatic melanomas to urinary bladder used to be mainly autopsy findings [1, 11] and currently, apart from case reports and small case series, no comprehensive cohorts of patients with metastatic melanoma to the urinary bladder have been reported [3, 12]. Both the great rarity and the limited literature references make metastatic melanomas to urinary bladder a serious diagnostic challenge for histopathologists and raise severe diagnostic issues on the differential diagnosis from other urinary bladder neoplasms. The determination of the original focus of metastatic melanomas to urinary bladder is convenient in some cases, where the primary focus is already known from patient's reported clinical history. However, when two independent primary foci of malignant melanoma coexist or have already been manifested, the determination of origin is a challenge by itself and exceedingly difficult to correlate the separate histological findings. The contribution of contemporary methods of molecular pathology is considered more than necessary.

In the presenting case, the persisting diagnostic challenge is discussed as a potential association of a recent melanocytic lesion in the urinary bladder with either a cutaneous melanoma which occurred seven years ago or an ocular melanoma which occurred twenty-five years ago. The possibility of correlation between all these three involved anatomic sites cannot be easily rejected.

## 2. Case Presentation

A 77-year-old male had been diagnosed with ocular melanoma 25 years ago and had been subjected to radiotherapy. Seven years ago, a cutaneous lesion appeared and it had been identified as malignant cutaneous melanoma. In both occasions, diagnostic and therapeutic procedures were performed at different hospitals. The associated histopathology report which referred to ocular melanoma was not available in patient's medical record. However, the previous histopathology report, relative to cutaneous melanoma, was provided.

**Figure 1:**
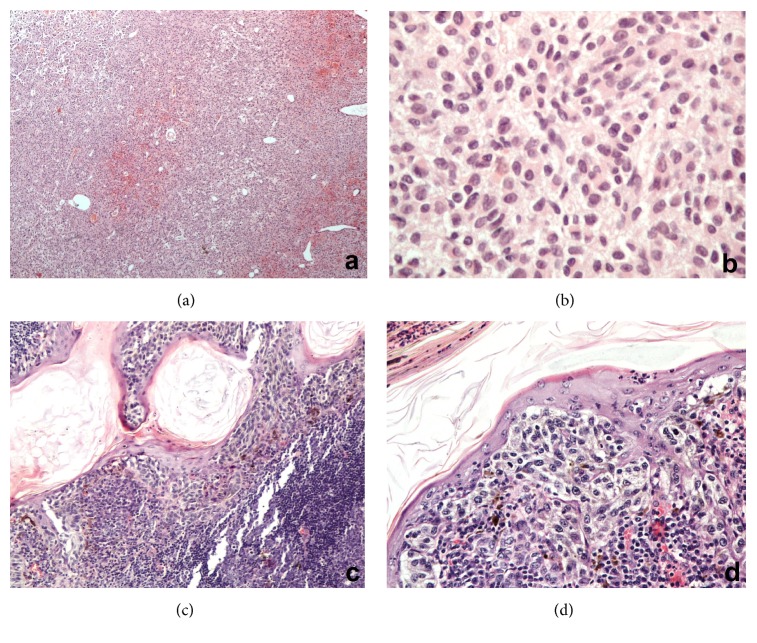
Histopathologic features of the urinary bladder tumor (a, b) and the cutaneous lesion (c, d).

**Figure 2:**
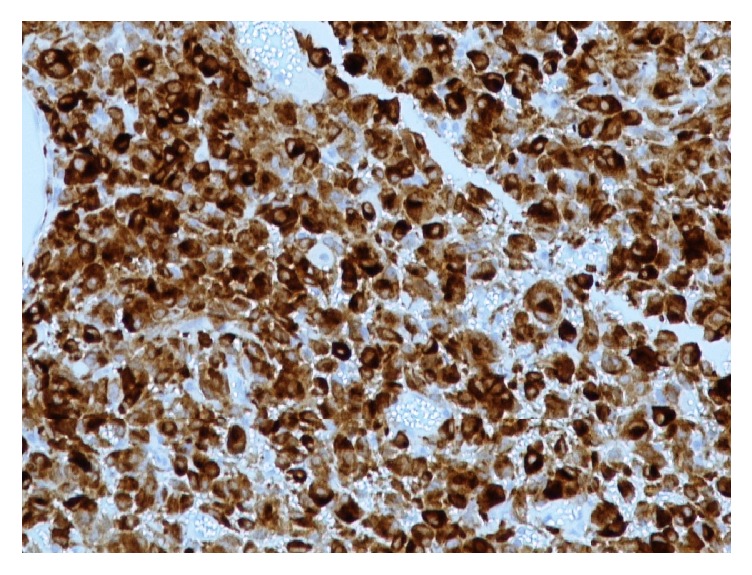
Melan A immunopositivity in the cells of the TURBT specimen.

The patient underwent transurethral resection (TURBT) of a polypoid lesion in urinary bladder which resected all the apparent gross lesion with gross total resection of 3 cm^3^ tumor volume. During microscopic examination of the TURBT specimen, the presence of malignant tumor segments was ascertained, which were characterized by high cellular density areas. The cytological features of tumor cells included large nuclei with variation in size and prominent nucleoli (Figures 1(a) and 1(b)). Immunohistochemical expressions (Table 1) of S100 protein, Melan A (Figure 2), HMB45, and Vimentin were positive, while expressions of Cytokeratin AE1/AE3, Desmin, Chromogranin, and CD99 were negative. It is worth mentioning that in the examined specimen there were detected areas of normal mucosa (transitional epithelium) with neither dysplastic lesions nor in situ carcinoma.

According to the available previous histopathology report, in relation to the cutaneous melanoma which occurred seven years ago, the diagnosis was the one of superficial spreading melanoma. The surgical specimen received at that time was a skin ellipse measuring 2.6 × 1.4 × 0.4 cm with raised, pigmented lesion measuring 1,7 cm in greater dimension. More specifically, there was a malignant melanocytic lesion mainly in horizontal growth phase with focal vertical growth, which infiltrated the papillary dermis, while Breslow thickness was estimated at 0.45 mm and evaluated as Clark's level III (Figures 1(c) and 1(d)). Immunohistochemical markers were positive for S100, Melan A, and HMB45 and negative for Cytokeratins and EMA. The lesion was excised within healthy tissues.

Afterwards and subsequent to the initial specimen examination, consultation was requested and a paraffin block of the TURBT specimen was sent for secondary opinion and further examination due to the probable correlation between the aforementioned melanocytic tumors and the recent bladder tumor. According to the consultation report, there was concordance over the morphological and the immunohistochemical findings of the initial examination. The existence of melanoma in vertical phase growth was also confirmed with necrotic areas and considerable ulcerative surface while Breslow thickness was estimated at least 0.55 mm. The neoplastic cell population was dominated by epithelioid type cells with focally severe atypia which conveyed plasmacytoid features. In some areas, single giant, multinucleated, or binucleated cellular forms were also seen with sporadic intranuclear inclusions. The MIB1/Ki-67 index exhibited a wide range from 3% to 15%. At the same time, genetic analysis was performed for the BRAF, GNA11, GNAQ, and C-KIT mutation status. The genetic analysis proved positive for the exon 4 c.548G>A p.R183Q GNAQ mutation while it provided negative results for the rest of all.

## 3. Discussion

The diagnostic challenge lied in the probable correlation between the three melanocytic lesions which occurred in three different anatomic sites. Despite the fact that urinary bladder constitutes an extremely uncommon metastasis site for both ocular and cutaneous malignant melanomas [13–16], the patient had remained free of metastatic disease for an interval of 25 years. This timeframe is considered to be too long for the emergence of distant metastasis [17, 18]. The epithelioid cells detected in the TURBT specimen exhibited some morphological features similar to those of epithelioid type of uveal malignant melanoma, but given that the specific histological type predicts more aggressive behavior [19], the metastasis free interval of 25 years could not be easily justified. Nevertheless, it has been reported that ocular melanoma may exhibit metastasis free intervals longer than 25 years [20, 21].

Intraocular melanoma may occur at any point of the uveal tract. Patient survival is directly associated with the histological type of the primary ocular focus, which is designated as the most important parameter, while the survival rate seems to be higher for spindle cell type in contrary to epithelioid type [19]. It is also worth mentioning that some researchers classify spindle cell melanoma as uveal nevus [22]. According to existing literature, additional prognostic features are tumor size at primary focus, intraocular localization, optic nerve involvement, tumor necrosis presence or absence, tumor neovascularization, and lymphocytic infiltration, as well as nuclear features of neoplastic cell population [23–25]. By order of priority, uveal melanoma gives metastases to liver, lungs, bones, and skin [14, 23]. According to the literature data, manifestation of distant metastases varies and their occurrence may emerge even after many years since eye enucleation and supplementary radiotherapy or/and chemotherapy [20, 21]. Furthermore, existing evidence regarding metastasis from ocular melanoma with morphological features simulating blue nevi [26] made the diagnostic challenge even more complicated. In order to get more insight about the physical course of metastasizing uveal melanomas to the urinary bladder, we performed a literature search for previously published cases. Only four cases have been reported so far, including the present case. All of them included patients of older age, who presented with urinary bladder metastases from 6 to 25 years after the initial diagnosis of uveal melanoma (Table 2).

The molecular examination proved to be of paramount importance for making the correct diagnosis. According to the best of our knowledge, this is the first case report which used molecular examination to distinguish whether the primary focus of a metastatic lesion of the urinary bladder was ocular or cutaneous. In fact, it has been suggested that GNAQ mutations are an early event characterizing over 80% of uveal melanomas [30, 31]. Therefore, the presence of that specific mutation in the material retrieved from the TURBT specimen confirmed the fact that the metastasis to the urinary bladder originated from the uveal melanoma diagnosed 25 years ago. At the time of writing (five months after TURBT), the patient has been diagnosed with multiple metastases to liver and spleen, as well as to celiac and mediastinal lymph nodes.

## Figures and Tables

**Table 1:** Panel of antibodies used in the present case report. RTU: ready to use.

Antibody	Clone	Company	Dilution
Anti-Melan A	A103	BioGenex, USA	RTU
Anti-S100	AR058-10R	BioGenex, USA	RTU
Anti-Melanosome	HMB45	Zeta Corporation, USA	1 : 100
Anti-Vimentin	V9	BioGenex, USA	RTU
Anti-Desmin	DE-R-11	Leica Biosystems, UK	RTU
Anti-Chromogranin A	5H7	Leica Biosystems, UK	RTU
Anti-EMA	GP1.4	Thermo Fisher Scientific, UK	RTU
Anti-CD99	12E7	Leica Biosystems, UK	RTU
Anti-Keratin	AE1/AE3	Zeta Corporation, USA	1 : 100

**Table 2:** Clinical features of the 4 cases of uveal melanoma metastases in the urinary bladder that have been reported so far in the literature.

Publication	Age	Gender	Concurrent metastases	Years after initial uveal melanoma diagnosis	Clinical course
Moore et al. [27]	82	Male	None	12	Free of disease at 9 months
Irisawa et al. [28]	77	Male	Widespread	6	Death at 10 months
Wisenbaugh et al. [29]	62	Female	Widespread	10	Death at 5 months
Present case	77	Male	Widespread	25	Widespread multiple metastases at months
